# Cellular model of neuronal atrophy induced by DYNC1I1 deficiency reveals protective roles of RAS-RAF-MEK signaling

**DOI:** 10.1007/s13238-016-0301-6

**Published:** 2016-08-10

**Authors:** Zhi-Dong Liu, Su Zhang, Jian-Jin Hao, Tao-Rong Xie, Jian-Sheng Kang

**Affiliations:** 1 Key Laboratory of Nutrition and Metabolism, Institute for Nutritional Sciences, Shanghai Institutes for Biological Sciences, Chinese Academy of Sciences, Shanghai, 200231 China; 2 University of Chinese Academy of Sciences, Beijing, 100049 China

**Keywords:** RAS-RAF-MEK pathway, atrophy, dynein intermediate chain, mitochondria, hippocampal neuron, autophagy

## Abstract

Neuronal atrophy is a common pathological feature occurred in aging and neurodegenerative diseases. A variety of abnormalities including motor protein malfunction and mitochondrial dysfunction contribute to the loss of neuronal architecture; however, less is known about the intracellular signaling pathways that can protect against or delay this pathogenic process. Here, we show that the DYNC1I1 deficiency, a neuron-specific dynein intermediate chain, causes neuronal atrophy in primary hippocampal neurons. With this cellular model, we are able to find that activation of RAS-RAF-MEK signaling protects against neuronal atrophy induced by DYNC1I1 deficiency, which relies on MEK-dependent autophagy in neuron. Moreover, we further reveal that BRAF also protects against neuronal atrophy induced by mitochondrial impairment. These findings demonstrate protective roles of the RAS-RAF-MEK axis against neuronal atrophy, and imply a new therapeutic target for clinical intervention.

**Electronic supplementary material:**

The online version of this article (doi:10.1007/s13238-016-0301-6) contains supplementary material, which is available to authorized users.

## Introduction

For normal aging, age-related brain atrophy is a mild process, responsible for increased risk of memory decline with increasing age (Pakkenberg et al., 2003; Fox and Schott, 2004), whereas the highly accelerated rate of brain atrophy has a major or sole role in neurodegenerative diseases (Regeur et al., 1994; Schott et al., 2003). Loss of neuronal architecture is the main contributor to brain atrophy (Swaab et al., 1994; Freeman et al., 2008), thus atrophy of brain and neuron can be well defined as the shortening or shrinkage of neurites. Neuritic atrophy is a common pathological feature of many neurodegenerative disorders including amyotrophic lateral sclerosis (ALS), Alzheimer’s disease (AD), Parkinson’s disease (PD), and Huntington’s disease (HD). A neuron consists of a soma and long neurites including one axon and multiple dendrites. The physiological structures of neurons render them particularly vulnerable to motor protein malfunction, protein aggregation, and mitochondrial dysfunction. Particularly, abnormalities to dynein and mitochondria are linked to ALS (Soo et al., 2011).

**Figure 1:**
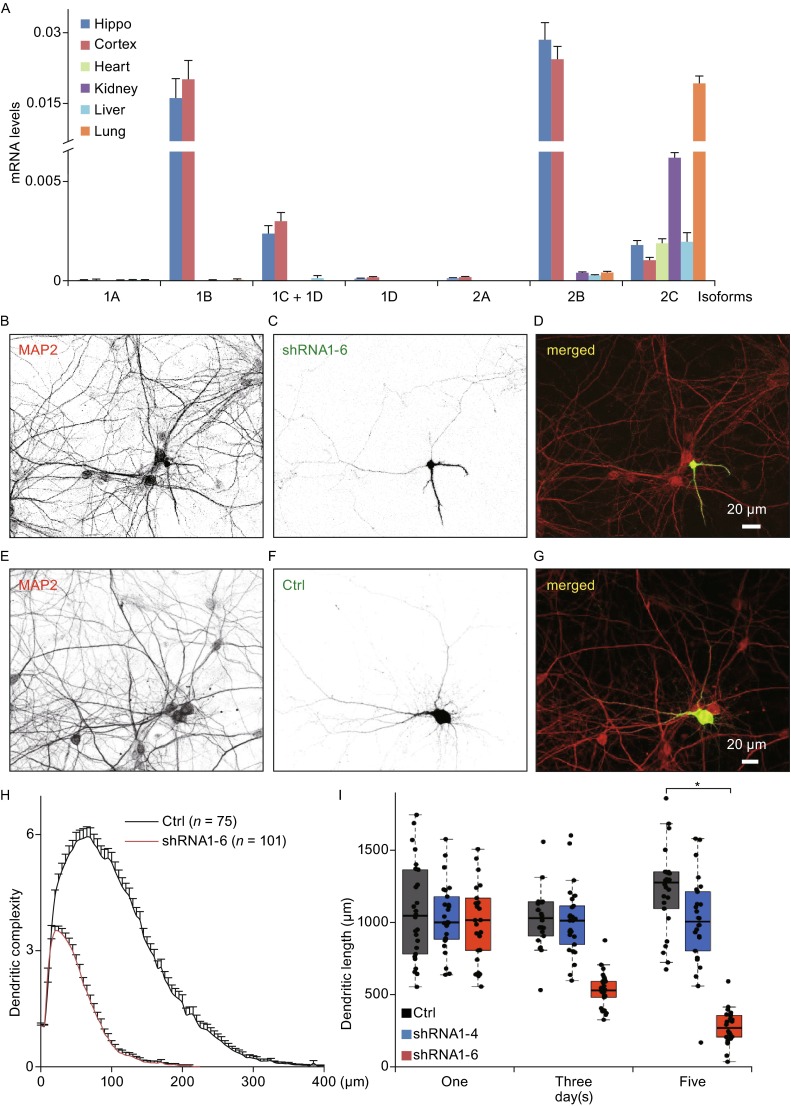
**Knockdown of cytoplasmic dynein 1 intermediate chain 1 (** ***Dync1i1*** **) causes dendritic atrophy in primary hippocampal neurons**. (A) The quantitative-PCR results show the expression levels of cytoplasmic dynein 1 intermediate chains, including the isoforms of *Dync1i1* (1A, 1B, 1C, and 1D) and the isoforms of *Dync1i2* (2A, 2B, and 2C) in P0 rat hippocampus, cortex, heart, kidney, liver, and lung tissues. (B–G) Representative neurons are transfected with shRNA1-6 (B–D, a specific shRNA of *Dync1i1*, see also Fig. S1) or Ctrl (E–G) at DIV6 and immunostained for the dendritic marker MAP2 (red in D and G) at DIV11. Green neurons are shRNA1-6 or Ctrl transfected neurons (green in D and G). The scale bars represent 20 μm. (H) Sholl analysis for dendritic complexity of neurons transfected with control vector (Ctrl, *n* = 75) and *Dync1i1* shRNA1-6 (*n* = 101). In control neurons, the maximum length of dendrite branches is found between 300 and 400 µm from the cell body. The shRNA1-6 transfected neurons show marked shortened dendrites such that the majority of dendrites were located within 200 µm of the soma. Data are represented as mean ± SEM. (I) Scatterplots with boxplots show that knockdown of DYNC1I1 expression causes dendritic atrophy. Primary hippocampal neurons are transfected with control vector (Ctrl, *n* = 26, gray box), control shRNA (shRNA1-4, *n* = 26, blue box) or shRNA1-6 (*n* = 26, red box) of *Dync1i1* at DIV6. Following transfection, neurons are cultured additional 1, 3 or 5 days before imaging and quantification of total dendritic length. The total dendritic lengths of control neurons slightly increase with increasing days *in vitro*. There is almost no length change of shRNA1-4 transfected neurons. However, the total dendritic lengths of shRNA1-6 transfected neurons are gradually and dramatically reduced at 3 and 5 days after transfection. *, *P* < 0.001

Cytoplasmic dynein is the main driving force for minus-end-directed transport of cargos (Holzbaur and Vallee, 1994). Cytoplasmic dynein is a large protein complex (~1.5 MDa) containing heavy chains, intermediate chains, light intermediate chains, and light chains (Pfister et al., 2005). Mutations and 9-bp deletion of cytoplasmic dynein heavy chain (*Dync1h1*) are sufficient to cause neuron degeneration (Hafezparast et al., 2003; Chen et al., 2007; Banks and Fisher, 2008; Lipka et al., 2013). Mutations of cytoplasmic dynein light intermediate chain 2 (*Dync1li2*) and dynein intermediate chain (*Dync1i*) result in reduction of dendrite arborization of *Drosophila* neurons (Zheng et al., 2008; Boylan and Hays, 2002). However, less is known about the physiologic role of *Dync1i* in higher animals, especially the isoforms of *Dync1i1*, which are neuron-specific and not expressed in glia in the brain (Myers et al., 2007). Two genes (*Dync1i1* and *Dync1i2*) in rodents encode cytoplasmic dynein intermediate chains, in which *Dync1i1* is a neuron-specific gene (Fig. 1A). In line with previous reports (Myers et al., 2007), most mRNAs of the intermediate chain isoforms are expressed in rat brain, while the mRNA of *Dync1i2C* and *Dync1i2B* are expressed in most tissues (Fig. 1A). Here, we demonstrate that the knockdown of *Dync1i1* causes neuronal atrophy and decreases mitochondrial motility in rat primary hippocampal neurons.

Using the cellular model of neuronal atrophy caused by DYNC1I1 deficiency, we are able to identify that RAS-RAF-MEK signaling, but not PI3K-AKT signaling, protects neurons against dendritic atrophy in primary hippocampal neurons, and reveals that RAS-RAF signaling activates MEK-dependent protective autophagy. Moreover, we further demonstrate that BRAF can also protect dendrites from atrophy arisen from mitochondrial dysfunction. These findings of the RAS-RAF-MEK pathway for neuronal atrophy protection may provide a therapeutic target against the on-sets of neuronal atrophy.

## Results

### Knockdown of DYNC1I1 expression causes dendritic atrophy of primary hippocampal neurons

Considering DYNC1I2 may play housekeeping function (Myers et al., 2007) and neurites of cultured neuron grow fast before 7 days *in vitro* (DIV7) (Dotti et al., 1988), we selectively knockdown DYNC1I1 expression at DIV6 in rat primary hippocampal neurons. Neurons transfected with shRNA1-6, a specific shRNA of *Dync1i1* (Fig. S1A–F), show reduced dendritic complexity and shortened dendritic length (Fig. 1B–D) compared to control neurons (Fig. 1E–G) at DIV11. For dendritic branches, the length of single branch seldom distributes beyond 350 μm in control neurons or 200 μm in DYNC1I1-knockdown neurons (Fig. 1H). Compared to the total dendritic lengths of control neurons (1100 ± 453 μm, *n* = 75, mean ± SD), the total dendritic lengths of DYNC1I1 knockdown neurons (303 ± 307 μm, *n* = 101, mean ± SD) are dramatically decreased (*P* < 0.001, *t* test) at DIV11 (Fig. 1I). Knockdown-resistant isoforms of *Dync1i1* can partially rescue the phenotype caused by shRNA1-6 (Fig. S1C–H). The data suggest that the functions of DYNC1I1 in neuron are nonredundant and necessary for the maintenance of neuronal architecture.

The potential effects of shRNA1-6 on dendritic development or dendritic atrophy can both explain the reduced total dendritic lengths of DYNC1I1 knockdown neurons. To determine the effect of shRNA1-6, we have checked and quantified total dendritic lengths at different time points (1, 3, and 5 days) after shRNA1-6 transfection. As shown in Fig. 1F, the distributions of total dendritic lengths show large variance. For 1, 3, and 5 days after transfection, the total dendritic lengths (in μm, mean ± SD) of control neurons are 1075 ± 330, 1130 ± 391, and 1307 ± 420, respectively; for control shRNA1-4 transfected neurons, the lengths are 1037 ± 230, 1010 ± 234, and 1006 ± 310; while the lengths of shRNA1-6 transfected neurons are 1041 ± 340, 535 ± 119, and 279 ± 113, respectively. These results are in line with reports that the dendritic architecture of neurons are mature at stage 4 (DIV4-7), while the maturation of synapses proceeds for following 2 weeks (stage 5) *in vitro* (Lalli, 2014). The data demonstrate that the DYNC1I1 knockdown by shRNA1-6 results in dendritic atrophy rather than dendritic-development retardation (Fig. 1F). Thus, to address the question of whether there is an intrinsic signaling pathway for protecting neuron from atrophy, dendritic atrophy caused by DYNC1I1 knockdown in primary hippocampal neuron is a good cellular model of neuronal atrophy arisen by motor protein dynein malfunction.

### BRAF protects against dendritic atrophy caused by DYNC1I1 knockdown in primary hippocampal neurons

**Figure 2:**
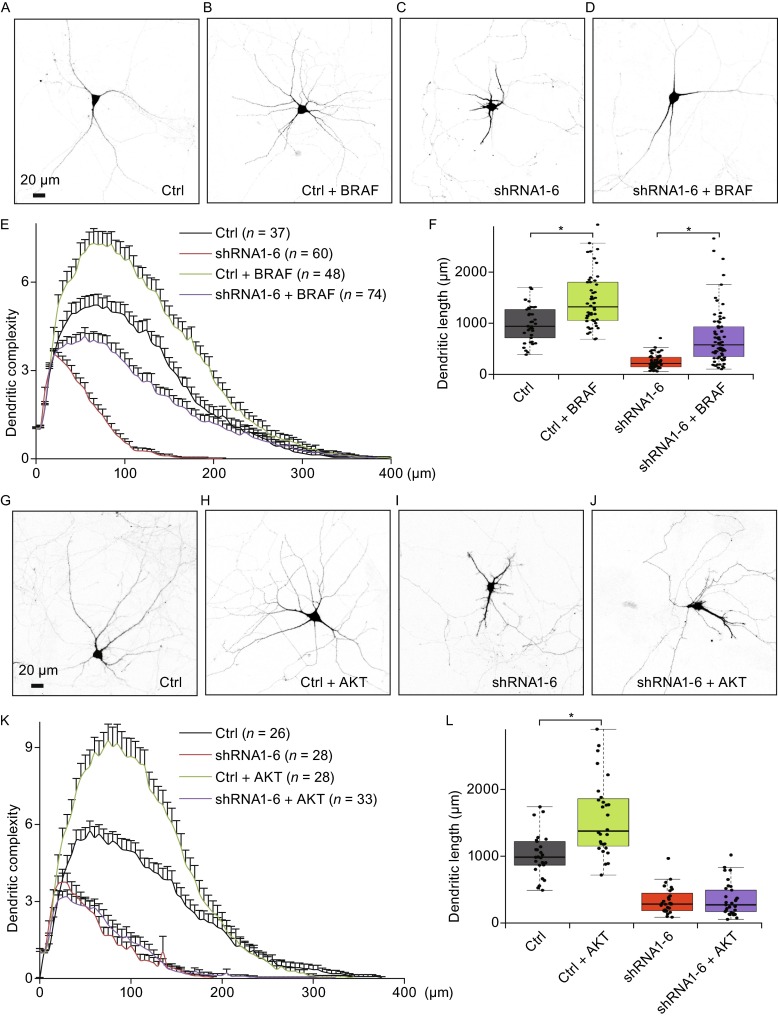
**BRAF but not AKT overexpression protects against dendritic atrophy caused by DYNC1I1 deficiency in primary hippocampal neurons**. (A–D) BRAF overexpression protects against dendritic atrophy caused by DYNC1I1 knockdown. Representative neurons are transfected with control vector (Ctrl) or shRNA1-6 co-transfected with or without BRAF at DIV6, and sequentially imaged at DIV11. The scale bars represent 20 μm. (E) Sholl analysis for dendritic complexity of neurons transfected with control vector combined without (Ctrl, *n* = 37, black) or with BRAF (Ctrl + BRAF, *n* = 48, green) or *Dync1i1* shRNA1-6 combined without (*n* = 60, red) or with BRAF (shRNA-6 + BRAF, *n* = 74, purple). Data are represented as mean ± SEM. (F) Scatterplots with boxplots show that BRAF overexpression promotes the dendritic growth of Ctrl neurons and protects dendritic atrophy caused by DYNC1I1 knockdown with shRNA1-6. (G–J) AKT overexpression has no effect on dendritic atrophy caused by DYNC1I1 knockdown. Representative neurons are transfected with control vector (Ctrl) or shRNA1-6 co-transfected with or without AKT at DIV6, and imaged at DIV11. The scale bars represent 20 μm. (K) Sholl analysis for dendritic complexity of neurons transfected with control vector combined without (Ctrl, *n* = 26, black) or with AKT (Ctrl + AKT, *n* = 28, green) or *Dync1i1* shRNA1-6 combined without (*n* = 28, red) or with AKT (shRNA-6 + AKT, *n* = 33, purple). Data are represented as mean ± SEM. (L) Scatterplots with boxplots show that AKT overexpression can also enhance the dendritic growth of Ctrl neurons, but fail to protect from dendritic atrophy caused by Dync1i1 knockdown with shRNA1-6. *, *P* < 0.001

For signaling pathways, we focus on the RAS-RAF-MEK-ERK mitogen activated protein kinase (MAPK) cascade and phosphoinositide 3-kinase (PI3K)-Protein kinase B (PKB, also known as AKT) pathways since they are both downstream signaling of neurotrophic factors (Chao, 2003). We overexpress AKT or BRAF in rat primary hippocampal neurons, where BRAF is the dominant functional RAF homolog in MAPK cascade and brain among three mammalian RAF proteins (ARAF, BRAF, and CRAF) (Galabova-Kovacs et al., 2006; Zhong et al., 2007). Consistent with the role in the signaling of neurotrophic factors, we have found that both BRAF and AKT overexpression can promote dendritic growth in control neurons (Fig. 2). The total dendritic lengths (in μm, mean ± SD) of control (Fig. 2A) and BRAF overexpressed neurons (Fig. 2B) are 1007 ± 342 and 1491 ± 540, respectively (Fig. 2F, gray and green boxes, *P* < 0.001, *t* test), and the lengths of control (Fig. 2G) and AKT overexpressed neurons (Fig. 2H) are 1021 ± 323 and 1581 ± 567, respectively (Fig. 2L, gray and green boxes, *P* < 0.001, *t* test).

However, only BRAF can partially rescue dendritic complexity and protect against dendritic atrophy caused by DYNC1I1 knockdown (Fig. 2D–F), and the total dendritic lengths (in μm, mean ± SD) of DYNC1I1-knockdown neurons without (Fig. 2C) or with BRAF overexpression (Fig. 2D) are 264 ± 135 and 776 ± 607, respectively (Fig. 2F, red and purple boxes, *P* < 0.001, *t* test). Meanwhile, AKT overexpression has no effect on dendritic complexity and atrophy (Fig. 2J–L), and the total dendritic lengths (in μm, mean ± SD) of DYNC1I1-knockdown neurons without (Fig. 2I) or with AKT overexpression (Fig. 2J) are 330 ± 197 and 347 ± 242, respectively (Fig. 2L, *P* = 0.78, *t* test). Thus, the results suggest that RAF signaling may have important role in protecting against neuronal atrophy caused dynein malfunction.

### RAS-RAF-MEK signaling protects against dendritic atrophy by activating MEK-dependent autophagy

**Figure 3:**
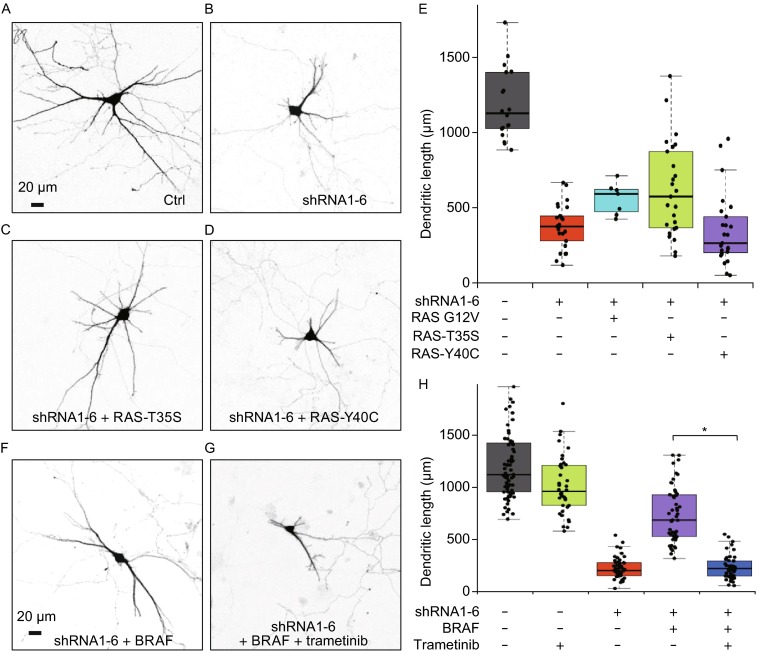
**RAS-RAF-MEK signaling pathway protects against dendritic atrophy caused by DYNC1I1 deficiency**. (A–D) Dominant active mutant G12V of RAS and selective RAF-activator mutant (G12V, T35S) of RAS protect against dendritic atrophy caused by DYNC1I1 knockdown, while selective AKT-activator mutant (G12V, Y40C) of RAS has no effect. Primary hippocampal neurons are transfected with control vector (Ctrl) or shRNA1-6 co-transfected with RAS-G12V, RAS-G12V-T35S or RAS-G12V-Y40C at DIV6, and imaged at DIV11. The scale bars represent 20 μm. (E) Scatterplots with boxplots show the total dendritic length distribution of neurons transfected with control vector (Ctrl, *n* = 18, gray), shRNA1-6 (*n* = 25, red), shRNA1-6 with RAS-G12V (*n* = 7, light blue), RAS-G12V-T35S (RAS-T35S, *n* = 25, green) or RAS-G12V-Y40C (RAS-Y40C, *n* = 25, purple). (F and G) Trametinib, a specific inhibitor of MEK kinase in RAS-RAF-MEK signaling, inhibits the protective role of BRAF in dendritic atrophy caused by DYNC1I1 deficiency with shRNA1-6. Neurons are co-transfected with BRAF and shRNA1-6 at DIV6, and imaged at DIV11. The scale bars represent 20 μm. (H) Scatterplots with boxplots show the total dendritic length distribution of neurons transfected with control vector treated without (*n* = 69, gray) or with 100 nmol/L of trametinib (*n* = 40, green), shRNA1-6 (*n* = 50, red), shRNA1-6 co-transfected with BRAF treated without (*n* = 50, purple) or with 100 nmol/L of trametinib (*n* = 50, blue). *, *P* < 0.001

To determine the signaling pathway for protecting dendritic atrophy, we use H-RAS mutants that activate RAF-MEK or/and PI3K-AKT signaling selectively or non-selectively. Dominant active mutant RAS-G12V activates both RAF-MEK and PI3K-AKT signaling, while RAS-G12V-T35S mutant selectively activates RAF-MEK and the RAS-G12V-Y40C mutant selectively stimulates PI3K-AKT (Fiordalisi et al., 2001). In line with the above results of BRAF and AKT (Fig. 2), we have found that RAS-G12V-T35S mutant but not RAS-G12V-Y40C mutant has similar protective effect of BRAF on dendritic atrophy caused by DYNC1I1 knockdown (Fig. 3A–E). The total dendritic lengths (in μm, mean ± SD) of control (Fig. 3A), shRNA1-6 (Fig. 3B), RAS-G12V-T35S (Fig. 3C), and RAS-G12V-Y40C (Fig. 3D) transfected neurons are 1188 ± 228, 377 ± 148, 623 ± 309, and 351 ± 234, respectively (Fig. 3E). Compared to the length of shRNA1-6 group, the lengths of RAS-G12V-T35S group are improved (*P* < 0.01, *t* test); RAS-G12V also plays protective role in dendritic atrophy by DYNC1I1 knockdown (562 ± 97 μm, mean ± SD, *P* < 0.01, *t* test), while RAS-G12V-Y40C has no effect (*P* = 0.64, *t* test). These results suggest RAS-RAF pathway rather than PI3K-AKT signaling can play protective role in neuronal atrophy caused by motor protein malfunction.

To confirm the role of RAS-RAF signaling in neuronal atrophy, we inhibit RAS-RAF-MEK signaling with pharmacological inhibitor trametinib, a specific inhibitor of MEK kinase (Yamaguchi et al., 2011). As shown in Fig. 3F–H, 100 nmol/L of trametinib blocks the protective effect of BRAF in dendritic atrophy induced by DYNC1I1 knockdown (*P* < 0.001, *t* test). For DYNC1I1-knockdown neurons at DIV11, the total dendritic lengths (μm, mean ± SD) of BRAF overexpressed neurons without (Fig. 3F) or with 100 nmol/L of trametinib treatment (Fig. 3G) are 740 ± 265 and 234 ± 112, respectively (Fig. 3H, purple and blue boxes), where the length of trametinib treated neurons with BRAF overexpression does not differ from the length of shRNA1-6 transfected neurons (224 ± 102 μm, mean ± SD, *P* = 0.67, *t* test). Meanwhile, 100 nmol/L of trametinib slightly reduces the total dendritic lengths of control neurons (Fig. 3H, gray and green boxes), where the lengths (in μm, mean ± SD) of neurons treated with or without trametinib are 1016 ± 268 and 1220 ± 355, respectively (*P* < 0.01, *t* test). Together, these results indicate that RAS-RAF-MEK signaling can play protective a role in dendritic atrophy caused by dynein malfunction.

### BRAF protects against DYNC1I1 deficiency induced dendritic atrophy by activating MEK-dependent autophagy

**Figure 4:**
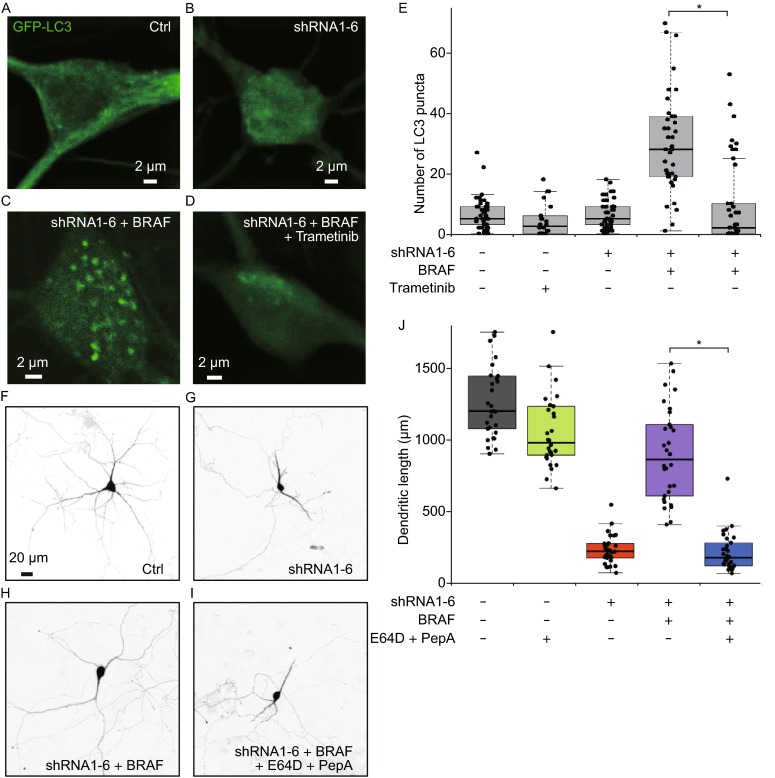
**BRAF overexpression protects against DYNC1I1 deficiency induced dendritic atrophy by activating MEK-dependent autophagy**. (A–D) BRAF overexpression enhances protective autophagy, which is MEK dependent. Primary hippocampal neurons are co-transfected GFP-LC3 with blank vector, shRNA1-6, shRNA1-6 and BRAF treated without or with 100 nmol/L of MEK inhibitor trametinib at DIV6, cultured additional 5 days and imaged at DIV11. Autophagosomes (green puncta) are labeled with GFP-LC3. All photos of GFP-LC3 are imaged with confocal microscope and further deconvolved for clarity. The scale bars represent 2 μm. (E) Scatterplots with boxplots show the number distribution of GFP-LC3 puncta in the soma of neurons transfected with control vector treated without (*n* = 41) or with 10 nmol/L of trametinib (*n* = 22), shRNA1-6 (*n* = 48), shRNA1-6 co-transfected with BRAF treated without (*n* = 39) or with 100 nmol/L of trametinib (*n* = 44). (F–I) Lysosomal protease inhibitors (E64D and pepstatin A) can inhibit the protective role of BRAF in dendritic atrophy caused by DYNC1I1 knockdown with shRNA1-6. Neurons are transfected and treated without or with 1 μmol/L of E64D and pepstatin A at DIV6, cultured additional 5 days and imaged at DIV11.The scale bars represent 20 μm. (J) Scatterplots with boxplots show the total dendritic length distribution of neurons transfected with control vector treated without (*n* = 29, gray) or with 1 μmol/L of E64D and pepstatin A (*n* = 30, green), shRNA1-6 (*n* = 30, red), shRNA1-6 co-transfected with BRAF treated without (*n* = 30, purple) or with 1 μmol/L of E64D and pepstatin A (*n* = 30, blue). *, *P* < 0.001

Since dynein is required for autophagic clearance (Maday et al., 2012; Kimura et al., 2008; Ravikumar et al., 2005), dynein malfunction may cause protein aggregation and mitochondrial dysfunctions, and result in neuronal atrophy. Thus, we have checked whether RAS-RAF-MEK signaling can enhance autophagic function against atrophy in neuron. Indeed, the number of autophagosomes labeled with GFP-LC3 is dramatically increased in DYNC1I1-knockdown neurons with BRAF overexpression (32 ± 19, Fig. 4C and 4E), which is blocked by MEK inhibitor trametinib (9 ± 14, *P* < 0.001, *t* test, Fig. 4D and 4E). Whereas, the numbers of autophagosomes in control neurons treated without (Fig. 4A) or with trametinib, and DYNC1I1-knockdown neurons (Fig. 4B) are 7 ± 6, 5 ± 5, and 6 ± 5, respectively (*P* = 0.25, one-way ANOVA test, Fig. 4E). These results suggest BRAF can activate MEK-dependent autophagy (Fig. 4C), which is necessary for the protective role of BRAF in DYNC1I1 deficiency caused neuronal atrophy (Fig. 3F–H). Moreover, the decreased number of autophagosomes in the presence of MEK inhibitor trametinib indicates that BRAF increases MEK-dependent autophagic influx and activity.

Additionally, to test whether the activity of increased autophagosomes is necessary for BRAF to play protective role in neuronal atrophy, we used lysosomal protease inhibitors (E64D and pepstatin A) to block autophagic clearance (Klionsky et al., 2012). As shown in Fig. 4F–J, blocking autophagic clearance inhibits the protective role of BRAF in neuronal atrophy. Again, BRAF shows robust role of protection in dendritic atrophy caused by DYNC1I1 knockdown. The total dendritic lengths (in μm, mean ± SD) of DYNC1I1-knockdown neurons with or without BRAF overexpression are 910 ± 322 and 233 ± 101, respectively (Fig. 4F–J). The protective role of BRAF in neuronal atrophy is blocked by 1 μmol/L of E64D and pepstatin A treatment after transfection (218 ± 135 μm, mean ± SD, *P* < 0.001 compared to BRAF protected neurons, *P* = 0.62 compared to shRNA1-6 transfected neurons, *t* test, Fig. 4I and 4J). Thus, the results demonstrate that the activity of increased autophagosomes is necessary for the protective role of BRAF in neuronal atrophy. 1 μmol/L of inhibitors alone (E64D and pepstatin A) slightly reduces the total dendritic lengths of control neurons (Fig. 4J, green boxes), where the lengths (in μm, mean ± SD) of neurons treated with or without inhibitors are 1097 ± 312 and 1314 ± 317, respectively (*P* = 0.01, *t* test, Fig. 4J). The protective role of enhancing autophagic activity is also demonstrated with mTOR inhibitor rapamycin (Fig. S2), which also increase the number of autophagosomes in neurons (Fig. S3).

Lysosomal inhibitors can also increase the number of autophagosomes by blocking the function of lysosomal protease (Fig. S3F and S3G), but result in slightly reduced dendritic lengths (Fig. 4J). In addition, lysosomal inhibitors can slightly but not significantly increase the number of autophagosomes in BRAF protected shRNA1-6 transfected neurons (Fig. S3G), and block the protective role of BRAF in neuronal atrophy, which supports that lysosomal inhibitors block the activity of lysosomal enzymes to increase the number of autophagosomes in neurons. Whereas, BRAF increases the number of autophagosomes (Fig. 4C and 4E), enhances the activity of autophagy, and protects neuronal atrophy (Figs. 2D, 3F, and 4F), which are both MEK dependent and blocked by MEK inhibitor trametinib (Figs. 4D and 3G). Together, we reveal the protective function of RAS-RAF signaling in neuronal atrophy is mediated by activating MEK-dependent autophagy, which is protective and helpful against neuronal atrophy by cleaning protein aggregations and dysfunctional organelles, such as mitochondria.

### BRAF also protects against dendritic atrophy caused by mitochondrial dysfunction in primary hippocampal neurons

**Figure 5:**
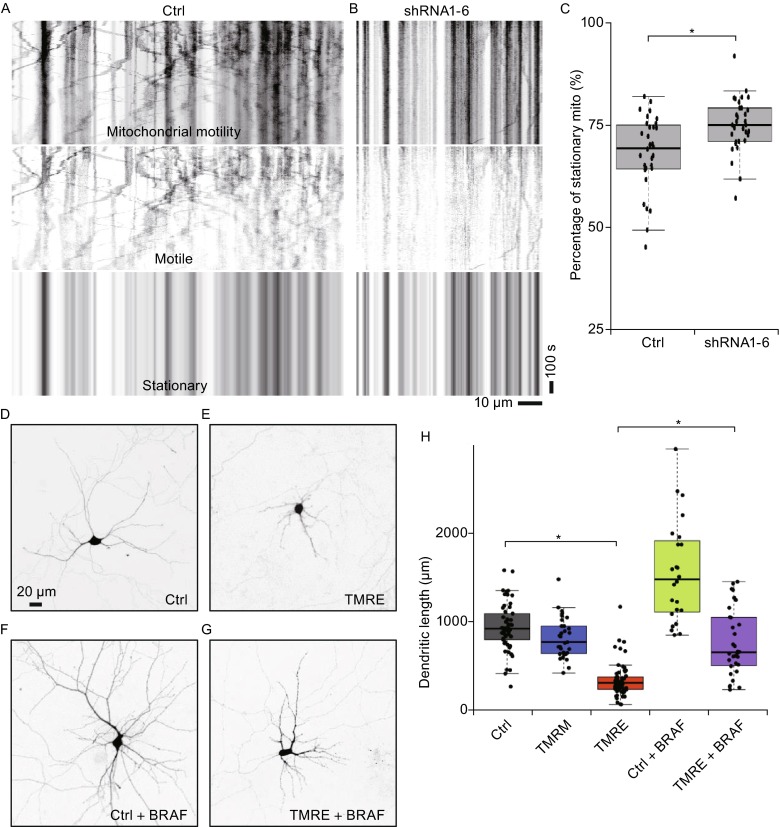
**BRAF overexpression also protects against dendritic atrophy caused by mitochondrial dysfunction in primary hippocampal neurons**. (A and B) Dync1i1 Knockdown decreases dendritic mitochondrial motility in primary hippocampal neurons. For clarity and quantification, motile and stationary dendritic mitochondria are separated with fast Fourier transform (FFT) algorithm. Kymographs show active mitochondrial motility in control neuron (A, Ctrl) and a few motile dendritic mitochondria in DYNC1I1-knockdown neuron (B, shRNA1-6). In kymographs, vertical lines represent stationary mitochondria, and slant lines or curves indicate motile mitochondria. Neurons are co-transfected at DIV6 with DsRed-mito and control vector (A) or shRNA1-6 (B), imaged at DIV11 and sequentially analyzed. The scale bars represent 10 μm (x axis) and 100 seconds (y axis). (C) Scatterplots with boxplots show the percentage distribution of stationary dendritic mitochondria in neurons transfected with control vector (*n* = 32) or shRNA1-6 (*n* = 32). (D–G) BRAF overexpression also protects against dendritic atrophy caused by mitochondrial dysfunction. Neurons are transfected with control vector (Ctrl) or BRAF treated with control solution or with 50 nmol/L of specific mitochondrial function inhibitor TMRE at DIV6, cultured additional 5 days and imaged at DIV11. The scale bars represent 20 μm. (H) Scatterplots with boxplots show the total dendritic length distribution of neurons transfected with control vector treated with vehicle (Ctrl, *n* = 53, gray), with 50 nmol/L of TMRM (*n* = 31, blue) or 50 nmol/L of TMRE (*n* = 63, red), or transfected with BRAF treated without (*n* = 24, green) or with 50 nmol/L of TMRE (*n* = 32, purple). *, *P* < 0.001

Considering dynein is the major motor protein for cargo transport in dendrites (Kapitein et al., 2010), especially for mitochondrial transport, we have checked the motility of dendritic mitochondria (Fig. 5A–C). As shown in the kymograph of Fig. 5A (See also supplemental video 1), dendritic mitochondria show active motility in control neuron, while only a few dendritic mitochondria are motile in DYNC1I1-knockdown neuron (Fig. 5B and supplemental video 2). To quantify the motility of dendritic mitochondria, motile and stationary dendritic mitochondria are separated with fast Fourier transform (FFT) algorithm (Fig. 5A and 5B). Considering motile mitochondria is prone to be ambiguous and overestimated due to the vague definition of motile mitochondria and the effect of photobleaching, we have quantified and compared the absolutely stationary mitochondria (The third row in Fig. 5A and 5B). The results demonstrate that stationary pool of dendritic mitochondria (75% ± 6%, mean ± SD in percentage) in DYNC1I1-knockdown neurons is significantly increased (versus 68% ± 9% in control neurons, *P* < 0.001, *t* test, Fig. 5A–C).

Illustrated in Fig. 5A–C, DYNC1I1 knockdown decreases mitochondrial motility, which may result in accumulation of dysfunctional mitochondria (Maday et al., 2012; Xie et al., 2015). In turn, the accumulation of dysfunctional mitochondria might be an important or major factor to cause neuronal atrophy. To inspect this, we used a specific inhibitor (tetramethylrhodamine ethyl ester, TMRE) of mitochondrial function (Scaduto and Grotyohann, 1999), which is a frequently used mitochondrial marker. As shown in Fig. 5D–J, compared to the total dendritic lengths (in μm, mean ± SD) of control neurons treated with relevant DMSO solution (944 ± 278, Fig. 5D and 5H) or a non-inhibitory mitochondrial marker (tetramethylrhodamine methyl ester, TMRM) at 50 nmol/L (Scaduto and Grotyohann 1999) (818 ± 234, Fig. 5H, blue box), neurons treated with 50 nmol/L of TMRE show shortened dendritic lengths (421 ± 229, *P* < 0.001, *t* test, Fig. 5E and 5H, red box). Thus, mitochondrial dysfunction is sufficient to result in neuronal atrophy.

Moreover, since both dynein and mitochondria are therapeutic targets in neurodegeneration (Eschbach and Dupuis, 2011; Banks and Fisher, 2008; Moreira et al., 2010), we are also wondering whether BRAF can protect against dendritic atrophy induced by mitochondrial dysfunction. For the total dendritic lengths (in μm, mean ± SD) of BRAF overexpressed neurons, BRAF promotes the dendritic growth of non-treated neurons (1570 ± 229, Fig. 5F and 5H), similar as shown in Fig. 2B and 2F; BRAF overexpressed neurons are somewhat resistant to TMRE treatment, and protect dendrites from atrophy induced by functional inhibition of mitochondria (775 ± 607, Fig. 5G and 5H, purple box). The total dendritic lengths of BRAF overexpressed neurons are significantly improved (*P* < 0.001, *t* test) compared to the lengths of TMRE treated control neurons (Fig. 5H red box). Together, these results based on above cellular models indicate that BRAF has a general protective role in neuronal atrophy caused by dynein malfunction or mitochondrial impairment.

## Discussion

Dynein is not only the major motor protein for dendritic cargo transport (Kapitein et al., 2010) and dendritic mitochondrial motility (Fig. 5), but also plays important roles in axonal structures and polarities (Song et al., 2009; Zheng et al., 2008). DYNC1I1 knockdown causes abnormal ER distribution in axon (Fig. S4), which is in line with the reported gatekeeper function of dynein (Song et al., 2009; Zheng et al., 2008). As an important cargo binding subunit of dynein (Ha et al., 2008), dynein intermediate chains (DYNC1I1 and DYNC1I2) are necessary for the functional integrity of dynein (Figs. 5 and S4) and the maintenance of neuronal architecture (Fig. 1). In addition, functions of both DYNC1I1 and DYNC1I2 are regulated by phosphorylation. The phosphorylation of DYNC1I1 serine 83 or DYNC1I2 serine 84 inhibits dynein intermediate chain binding to dynactin or paxillin (Vaughan et al., 2001; Rosse et al., 2012); while the phosphorylation of DYNC1I1 serine 80 or DYNC1I2 serine 81 is MAP kinase ERK1/2 dependent and can strengthen dynein activity in signaling cargos transport (Mitchell et al., 2012). In our study, we cannot exclude that the protective role of RAS-RAF-MEK signaling in neuronal atrophy may be partially due to upregulating the phosphorylation of DYNC1I2 serine 81 and thus partially compensate the dynein malfunction by DYNC1I1 knockdown, although we fail to detect any change of phosphorylation of DYNC1I2 serine 81 for control and DYNC1I1 knockdown (data not shown) in primary cultured hippocampal neuron, which may be explained by the low transfection efficiency of primary neuron and the expression of DYNC1I2C in glia.

### Protective role of RAS-RAF-MEK axis in neuronal atrophy caused by dynein malfunction

In corroboration with our results, transgenic activation of RAS in neurons promotes neuronal growth and protects from lesion-induced degeneration (Heumann et al., 2000), but the mechanism is unknown; in addition, selective activation of BRAF can provide neuroprotection both *in vitro* and *in vivo* although it is not MEK dependent (Chin et al., 2004). Here, we identify that RAS-RAF signaling protects neurons against dendritic atrophy arisen from dynein malfunction, which relies on MEK-dependent autophagy (Fig. 4). MEK-dependent autophagy can be either protective or destructive autophagy (Wang et al., 2009) in non-neuronal cells. The role of MEK-dependent autophagy in neuron is unknown yet. Trametinib treatment and BRAF in control neurons doesn’t affect the numbers of autophagosomes (Figs. 4 and Fig. S3E), which suggests that MEK-dependent autophagy has minor role in neuron under normal condition. However, we demonstrate that RAS-RAF pathway activates protective autophagy in primary hippocampal neuron with DYNC1I1 knockdown (Fig. 4), which is favorable for structural and functional integrity of neuron.

**Figure 6:**
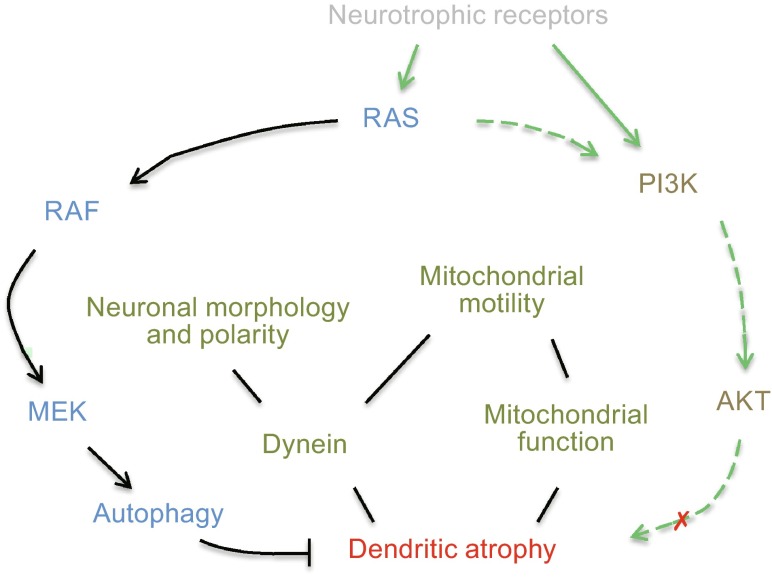
**Schematic summary of general roles of RAS-RAF-MEK pathway in protecting dendritic atrophy caused by dynein malfunction or mitochondrial dysfunction**

Both RAS-RAF and PI3K-AKT pathways are necessary for dendritic morphogenesis (Kumar et al., 2005) and neuron survival (Mazzoni et al. 1999). Additionally, PI3K-AKT signaling has a major role in antiapoptotic function (Brunet et al., 2001). Here, we demonstrate that only RAS-RAF-MEK pathway has the protective role in dendritic atrophy cause by DYNC1I1 knockdown (Fig. 6). Interestingly, it is recently reported that inhibition of RAS-MAPK pathway has a role in longevity of *Drosophila* (Slack et al., 2015). However, to avoid compromising brain function of higher animals (Fig. 2–4), our results raise caution about inhibiting RAS-RAF-MEK signaling for longevity. Thus, it deserves further studies on the roles of RAS-RAF-MEK signaling in longevity and neuronal atrophy with models of higher animals, such as rodents or monkeys.

### A more general role of RAS-RAF-MEK signaling in neuronal atrophy

In the past decades, dominated researches and drug developments have focused or based on cholinergic hypothesis or the amyloid cascade hypothesis for Alzheimer’s disease (Becker et al., 2008; Craig et al., 2011; Karran et al., 2011). However, the continual failures of clinical trials for neurodegenerative disorders suggest that it is important and necessary to think about new models and strategies, such as motor protein dynein malfunction and mitochondrial dysfunction, both of which are in the spotlight of neurodegeneration therapy, ALS in particular (Eschbach and Dupuis, 2011; Banks and Fisher, 2008; Moreira et al., 2010; Payne and Chinnery, 2015).

Here, based on the cellular models of neuronal atrophy, we demonstrate that BRAF has a general protective role in neuronal atrophy caused by dynein malfunction or mitochondrial impairment (Fig. 6). Dynein malfunction by DYNC1I1 knockdown decreases mitochondrial motility (Fig. 4), which may augment mitochondrial pathology. This study provides some missing linkages among dynein, mitochondria, and atrophy/degeneration. These findings about the RAS-RAF-MEK pathway for neuronal atrophy protection provide a therapeutic intervention signaling against the on-sets of neuronal atrophy caused by dynein malfunction or mitochondrial impairment. Importantly, neuronal atrophy is not only the hallmark of neurodegeneration, such as ALS, but also a continuous process in adult brain with increasing age (Pakkenberg et al., 2003; Fox and Schott, 2004). Therefore, more speculatively, it might even imply a potential target to ameliorate memory decline due to age-related brain atrophy.

## Materials and Methods

Additional information can be found in the supplemental materials and methods.

### Plasmids and shRNA

The isoforms of *Dync1i1* are cloned into the vector pEGFP-N1 with restriction sites *Eco*RI and *Bam*HI (NEB). The shRNA1-6 oligonucleotide for *Dync1i1* (5′-GCATGGAGCTGGTGTACAA-3′) and control shRNA1-4 oligonucleotide (5′-GCTGGAGCCAACCTTTCTT-3′) are constructed into the modified pSUPER vector (*Kan*^*r*^, EGFP expression) with *Bgl*II and *Hin*dIII restriction sites. The calcium phosphate method is used for transfection.

### Data and statistical analysis

Imaging is performed using an Olympus FV1000 confocal microscope with a 40×/0.95 objective (Olympus) for dendritic length imaging and a 60×/1.2w objective (Olympus) for high-resolution imaging at room temperature or mitochondrial motility study at 37°C. The sholl analysis of neuritic morphology and complexity is performed using Fiji/ImageJ software with Simple Neurite Tracer. Photos of GFP-LC3 labeled autophagosomes are imaged with confocal microscope and further deconvolved using the SharpStack Total Deconvolution function of Image-Pro Plus (Media Cybernetics). The numbers of autophagosomes are counted by triple-blinded analysis. Data sets in sholl-analysis graphs are presented as mean ± SEM from repeats in at least three independent experiments, while data sets in text are presented as mean ± SD. Scatterplots with boxplots are plotted with R software. For mitochondrial motility study, motile and stationary dendritic mitochondria are separated with an immobile filter, which is computed with FFT algorithm. When comparing multiple samples in a group, one-way ANOVA test is used. When comparing two samples, two-tailed Student’s *t* test is used.

## Electronic supplementary material

Below is the link to the electronic supplementary material.

Supplementary material 1 (PDF 634 kb)

Supplementary material 2 (MOV 1419 kb)

Supplementary material 3 (MOV 779 kb)

## References

[CR1] Banks GT, Fisher EM (2008). Cytoplasmic dynein could be key to understanding neurodegeneration. Genome Biol.

[CR2] Becker RE, Greig NH, Giacobini E (2008). Why do so many drugs for Alzheimer’s disease fail in development? Time for new methods and new practices?. J Alzheimers Dis.

[CR3] Boylan KLM, Hays TS (2002). The gene for the intermediate chain subunit of cytoplasmic dynein is essential in Drosophila. Genetics.

[CR4] Brunet A, Datta SR, Greenberg ME (2001). Transcription-dependent and -independent control of neuronal survival by the PI3K–Akt signaling pathway. Curr Opin Neurobiol.

[CR5] Chao MV (2003). Neurotrophins and their receptors: a convergence point for many signalling pathways. Nat Rev Neurosci.

[CR6] Chen X-J, Levedakou EN, Millen KJ, Wollmann RL, Soliven B, Popko B (2007). Proprioceptive sensory neuropathy in mice with a mutation in the cytoplasmic dynein heavy chain 1 gene. J Neurosci.

[CR7] Chin PC, Liu L, Morrison BE, Siddiq A, Ratan RR, Bottiglieri T, D’Mello SR (2004). The c-Raf inhibitor GW5074 provides neuroprotection in vitro and in an animal model of neurodegeneration through a MEK-ERK and Akt-independent mechanism. J Neurochem.

[CR8] Craig LA, Hong NS, McDonald RJ (2011). Revisiting the cholinergic hypothesis in the development of Alzheimer’s disease. Neurosci Biobehav Rev.

[CR9] Dotti CG, Sullivan CA, Banker GA (1988). The establishment of polarity by hippocampal neurons in culture. J Neurosci.

[CR10] Eschbach J, Dupuis L (2011). Cytoplasmic dynein in neurodegeneration. Pharmacol Ther.

[CR11] Fiordalisi JJ, Johnson RL, Ülkü AS, Der CJ, Cox AD, Der CJ, Balch WE (2001). Mammalian expression vectors for Ras family proteins: generation and use of expression constructs to analyze Ras family function. Methods in enzymology.

[CR12] Fox NC, Schott JM (2004). Imaging cerebral atrophy: normal ageing to Alzheimer’s disease. Lancet.

[CR13] Freeman SH, Kandel R, Cruz L, Rozkalne A, Newell K, Frosch MP, Hedley-Whyte ET, Locascio JJ, Lipsitz L, Hyman BT (2008). Preservation of neuronal number despite age-related cortical brain atrophy in elderly subjects without Alzheimer disease. J Neuropathol Exp Neurol.

[CR14] Galabova-Kovacs G, Kolbus A, Matzen D, Meissl K, Piazzolla D, Rubiolo C, Steinitz K, Baccarini M (2006). ERK and beyond: insights from B-Raf and Raf-1 conditional knockouts. Cell Cycle Georget. Tex.

[CR15] Ha J, Lo KW-H, Myers KR, Carr TM, Humsi MK, Rasoul BA, Segal RA, Pfister KK (2008). A neuron-specific cytoplasmic dynein isoform preferentially transports TrkB signaling endosomes. J Cell Biol.

[CR16] Hafezparast M, Klocke R, Ruhrberg C, Marquardt A, Ahmad-Annuar A, Bowen S, Lalli G, Witherden AS, Hummerich H, Nicholson S (2003). Mutations in dynein link motor neuron degeneration to defects in retrograde transport. Science.

[CR17] Heumann R, Goemans C, Bartsch D, Lingenhöhl K, Waldmeier PC, Hengerer B, Allegrini PR, Schellander K, Wagner EF, Arendt T (2000). Transgenic activation of Ras in neurons promotes hypertrophy and protects from lesion-induced degeneration. J Cell Biol.

[CR18] Holzbaur ELF, Vallee RB (1994). Dyneins: molecular structure and cellular function. Annu Rev Cell Biol.

[CR19] Kapitein LC, Schlager MA, Kuijpers M, Wulf PS, van Spronsen M, MacKintosh FC, Hoogenraad CC (2010). Mixed microtubules steer dynein-driven cargo transport into dendrites. Curr Biol.

[CR20] Karran E, Mercken M, Strooper BD (2011). The amyloid cascade hypothesis for Alzheimer’s disease: an appraisal for the development of therapeutics. Nat. Rev. Drug Discov..

[CR21] Kimura S, Noda T, Yoshimori T (2008). Dynein-dependent movement of autophagosomes mediates efficient encounters with lysosomes. Cell Struct Funct.

[CR22] Klionsky DJ, Abdalla FC, Abeliovich H, Abraham RT, Acevedo-Arozena A, Adeli K, Agholme L, Agnello M, Agostinis P, Aguirre-Ghiso JA (2012). Guidelines for the use and interpretation of assays for monitoring autophagy. Autophagy.

[CR23] Kumar V, Zhang M-X, Swank MW, Kunz J, Wu G-Y (2005). Regulation of dendritic morphogenesis by Ras–PI3K–Akt–mTOR and Ras–MAPK signaling pathways. J Neurosci.

[CR24] Lalli G (2014). Regulation of neuronal polarity. Exp Cell Res.

[CR25] Lipka J, Kuijpers M, Jaworski J, Hoogenraad CC (2013). Mutations in cytoplasmic dynein and its regulators cause malformations of cortical development and neurodegenerative diseases. Biochem Soc Trans.

[CR26] Maday S, Wallace KE, Holzbaur ELF (2012). Autophagosomes initiate distally and mature during transport toward the cell soma in primary neurons. J Cell Biol.

[CR27] Mazzoni IE, Saïd FA, Aloyz R, Miller FD, Kaplan D (1999). Ras regulates sympathetic neuron survival by suppressing the p53-mediated cell death pathway. J Neurosci.

[CR28] Mitchell DJ, Blasier KR, Jeffery ED, Ross MW, Pullikuth AK, Suo D, Park J, Smiley WR, Lo KW-H, Shabanowitz J (2012). Trk activation of the ERK1/2 kinase pathway stimulates intermediate chain phosphorylation and recruits cytoplasmic dynein to signaling endosomes for retrograde axonal transport. J Neurosci.

[CR29] Moreira PI, Zhu X, Wang X, Lee H, Nunomura A, Petersen RB, Perry G, Smith MA (2010). Mitochondria: a therapeutic target in neurodegeneration. Biochim Biophys Acta.

[CR30] Myers KR, Lo KW-H, Lye RJ, Kogoy JM, Soura V, Hafezparast M, Pfister KK (2007). Intermediate chain subunit as a probe for cytoplasmic dynein function: biochemical analyses and live cell imaging in PC12 cells. J Neurosci Res.

[CR31] Pakkenberg B, Pelvig D, Marner L, Bundgaard MJ, Gundersen HJG, Nyengaard JR, Regeur L (2003). Aging and the human neocortex. Exp Gerontol.

[CR32] Payne BAI, Chinnery PF (2015). Mitochondrial dysfunction in aging: much progress but many unresolved questions. Biochim. Biophys. Acta BBA - Bioenerg..

[CR33] Pfister KK, Fisher EMC, Gibbons IR, Hays TS, Holzbaur ELF, McIntosh JR, Porter ME, Schroer TA, Vaughan KT, Witman GB (2005). Cytoplasmic dynein nomenclature. J Cell Biol.

[CR34] Ravikumar B, Acevedo-Arozena A, Imarisio S, Berger Z, Vacher C, O’Kane CJ, Brown SDM, Rubinsztein DC (2005). Dynein mutations impair autophagic clearance of aggregate-prone proteins. Nat Genet.

[CR35] Regeur L, Badsberg Jensen G, Pakkenberg H, Evans SM, Pakkenberg B (1994). No global neocortical nerve cell loss in brains from patients with senile dementia of Alzheimer’s type. Neurobiol Aging.

[CR36] Rosse C, Boeckeler K, Linch M, Radtke S, Frith D, Barnouin K, Morsi AS, Hafezparast M, Howell M, Parker PJ (2012). Binding of dynein intermediate chain 2 to paxillin controls focal adhesion dynamics and migration. J Cell Sci.

[CR37] Scaduto RC, Grotyohann LW (1999). Measurement of mitochondrial membrane potential using fluorescent rhodamine derivatives. Biophys J.

[CR38] Schott JM, Fox NC, Frost C, Scahill RI, Janssen JC, Chan D, Jenkins R, Rossor MN (2003). Assessing the onset of structural change in familial Alzheimer’s disease. Ann Neurol.

[CR39] Slack C, Alic N, Foley A, Cabecinha M, Hoddinott MP, Partridge L (2015). The Ras-Erk-ETS-signaling pathway is a drug target for longevity. Cell.

[CR40] Song A, Wang D, Chen G, Li Y, Luo J, Duan S, Poo M (2009). A selective filter for cytoplasmic transport at the axon initial segment. Cell.

[CR41] Soo KY, Farg M, Atkin JD (2011). Molecular motor proteins and amyotrophic lateral sclerosis. Int J Mol Sci.

[CR42] Swaab DF, Hofman MA, Lucassen PJ, Salehi A, Uylings HBM (1994). Neuronal atrophy, not cell death, is the main hallmark of Alzheimer’s disease. Neurobiol Aging.

[CR43] Vaughan PS, Leszyk JD, Vaughan KT (2001). Cytoplasmic dynein intermediate chain phosphorylation regulates binding to dynactin. J Biol Chem.

[CR44] Wang J, Whiteman MW, Lian H, Wang G, Singh A, Huang D, Denmark T (2009). A non-canonical MEK/ERK signaling pathway regulates autophagy via regulating beclin 1. J Biol Chem.

[CR45] Xie Y, Zhou B, Lin M-Y, Wang S, Foust KD, Sheng Z-H (2015). Endolysosomal deficits augment mitochondria pathology in spinal motor neurons of asymptomatic fALS mice. Neuron.

[CR46] Yamaguchi T, Kakefuda R, Tajima N, Sowa Y, Sakai T (2011). Antitumor activities of JTP-74057 (GSK1120212), a novel MEK1/2 inhibitor, on colorectal cancer cell lines in vitro and in vivo. Int J Oncol.

[CR47] Zheng Y, Wildonger J, Ye B, Zhang Y, Kita A, Younger SH, Zimmerman S, Jan LY, Jan YN (2008). Dynein is required for polarized dendritic transport and uniform microtubule orientation in axons. Nat Cell Biol.

[CR48] Zhong J, Li X, McNamee C, Chen AP, Baccarini M, Snider WD (2007). Raf kinase signaling functions in sensory neuron differentiation and axon growth in vivo. Nat Neurosci.

