# Supplementary material for: Cellular model of neuronal atrophy induced by DYNC1I1 deficiency reveals protective roles of RAS-RAF-MEK signaling
Source: Protein Cell. 2016 Aug 10;7(9):638–50. doi: 10.1007/s13238-016-0301-6 (PMC5003791; doi:10.1007/s13238-016-0301-6)
Supplement: Supplementary file 1 — Supplementary material 1 (PDF 634 kb) [file 13238_2016_301_MOESM1_ESM.pdf]

I. Supplemental Figure Legends

Figure S1. Knockdown-resistant mutants of *Dync1i1* isoforms partially rescue dendritic atrophy in primary hippocampal neurons (Related to Figure 1).

(A) Knockdown efficiency of shRNAs for *Dync1i1*. COS7 cells are transfected with Dync1i1 and shRNA plasmids, and cultured for 2 days before Western Blotting. The expression of Dync1i1B-EGFP is effectively knockdowned by shRNA1-6, and less effectively by shRNA1-7 and shRNA1-5. As illustrated, shRNA1-4 has no knockdown effect, and used as control in Figure 1I. GFP is as a loading control. The expression of GFP is driven by CMV promotor, while shRNAs are driven by H1 promotor of modified pSuper construct (Methods).

(B) Specificity of shRNA1-6 for *Dync1i1*. The expression of Dync1i1B-EGFP in COS7 is effectively knockdowned by shRNA1-6, which has on effect on expression of DYNC1I2C-EGFP. Meanwhile, shRNA2-3 of *Dync1i2* specifically knockdowns the expression of DYNC1I2C-EGFP.

(C-F) All isoforms of *Dync1i1* are effectively knockdowned by shRNA1-6, while shRNA1-7 of *Dync1i1* is less effective. Mutants (1A_r, 1B_r, 1C_r and 1D_r) of *Dync1i1* isoforms are knockdown resistant, where "_r" means knockdown-resistant.

(G) Sholl analysis for dendritic complexity of neurons transfected with control vector (Ctrl, n=46, black), shRNA1-6 (n=51, red), shRNA1-6+1A_r (n=23, green), shRNA1-6+1B_r (n=18, purple), shRNA1-6+1C_r (n=30, blue) and shRNA1-6+1D_r (n=29, orange). Neurons are transfected at DIV6, imaged at DIV11 and sequentially quantified for total dendritic length. Data are represented as mean ± SEM.

(H) Scatterplots with boxplots show that knockdown-resistant mutants of *Dync1i1* isoforms partially rescue total dendritic length (the same datasets in panel F).

Figure S2. Rapamycin protects against dendritic atrophy caused by DYNC1I1 Knockdown in primary hippocampal neurons.

(A-C) Rapamycin (100 nM) protects against dendritic atrophy caused by DYNC1I1 Knockdown. Representive neurons are transfected with control vector (Ctrl) or shRNA1-6 at DIV6, and sequentially imaged at DIV11. The scale bars represent 20 μm.

(D) Sholl analysis for dendritic complexity of neurons transfected with control vector treated without (Ctrl, n=53, black) or with 100 nM of rapamycin (Ctrl+rapamycin, n=31, green) or *Dync1i1* shRNA1-6 treated without (n=48, red) or with 100 nM of rapamycin (shRNA-6+rapamycin, n=36, purple). Data are represented as mean ± SEM.

(E) Scatterplots with boxplots show that rapamycin can protect dendritic atrophy caused by DYNC1I1 Knockdown with shRNA1-6 (the same datasets in panel D). *, $p < 0.001$.

Figure S3. Autophagosome accumulation under various conditions.

(A-F) Autophagosome accumulation under various conditions. Primary hippocampal neurons are co-transfected GFP-LC3 with control vector, shRNA1-6, BRAF, shRNA1-6 and BRAF treated without or with 1 μM of E64D and pepstatin A, or 100 nM of rapamycin at DIV6, cultured additional 5 days and imaged at DIV11. Autophagosomes (green puncta) are labeled with GFP-LC3. All photos of GFP-LC3 are imaged with confocal microscope. The scale bars represent 5 μm.

(G) Scatterplots with boxplots show the number distribution of GFP-LC3 puncta in the soma of neurons transfected with BRAF (n=15, gray), control vector treated with 1 μM of E64D and pepstatin A (n=8, green), shRNA1-6 co-transfected with BRAF treated without (n=8, red) or with 1 μM of E64D and pepstatin A (n=14, purple), shRNA1-6 treated with 100 nM of rapamycin (n=27, blue).

Figure S4. Dync1i1 knockdown causes abnormal ER distribution in primary hippocampal neurons.

(A-H) ER is absent (pointed by arrow) in axonal initial segment (AIS), while ER shows abnormal distribution (indicated by arrow head) in AIS of Dync1i1 knockdown neuron with shRNA1-6. Neurons are co-transfected at DIV6 with DsRed-ER and blank vector (Ctrl, A-D) or shRNA1-6 (E-H), and immunostained for AIS marker Ankyrin G at DIV11. The scale bars represent 20 μm.

(I-J) For clarity, AIS regions are zoomed in. Dync1i1 knockdown causes abnormal ER distribution in the AIS of neurons, where ER is absent in control neurons. The scale bars represent 5 μm.

II. Supplemental Figures

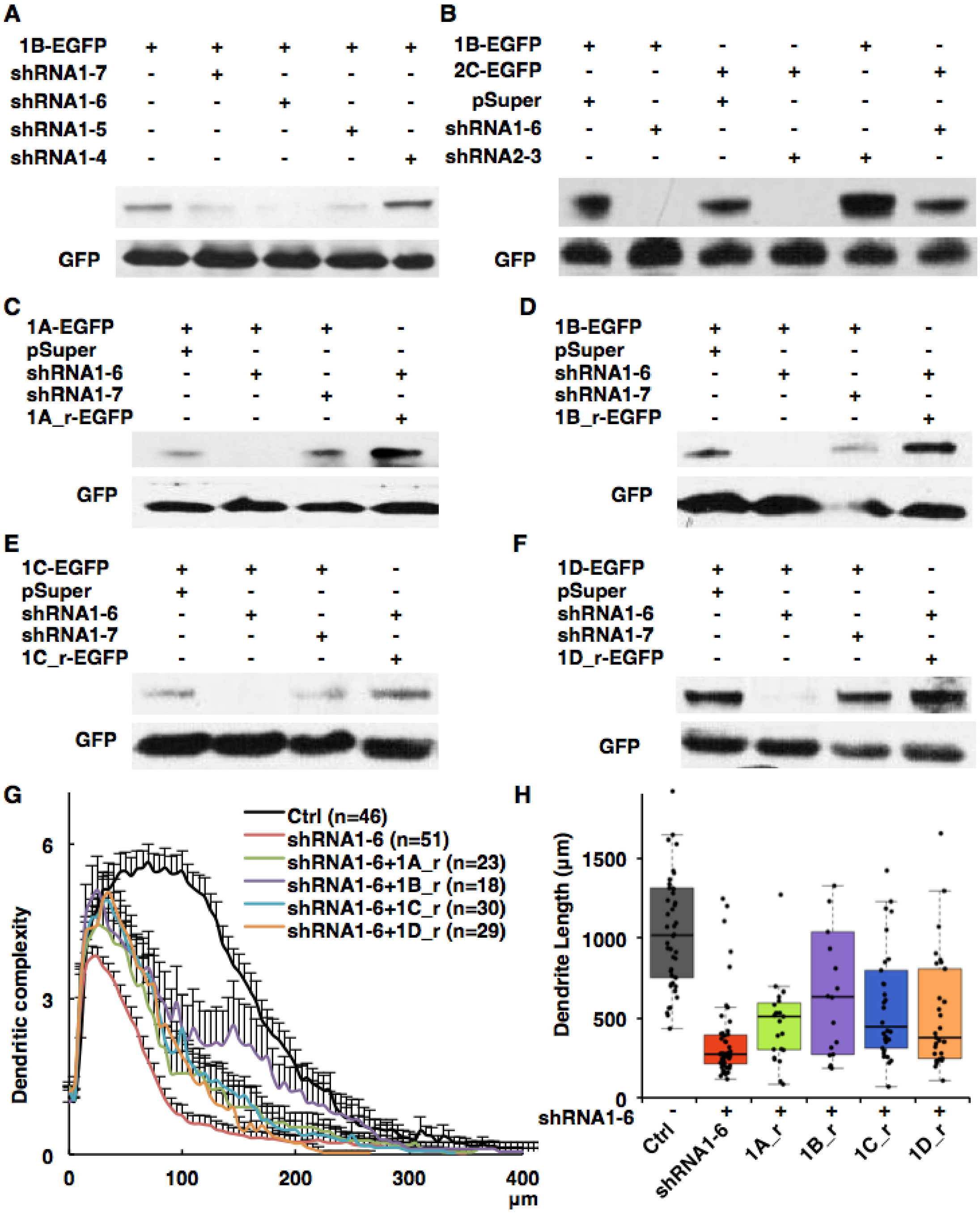

Figure S1.

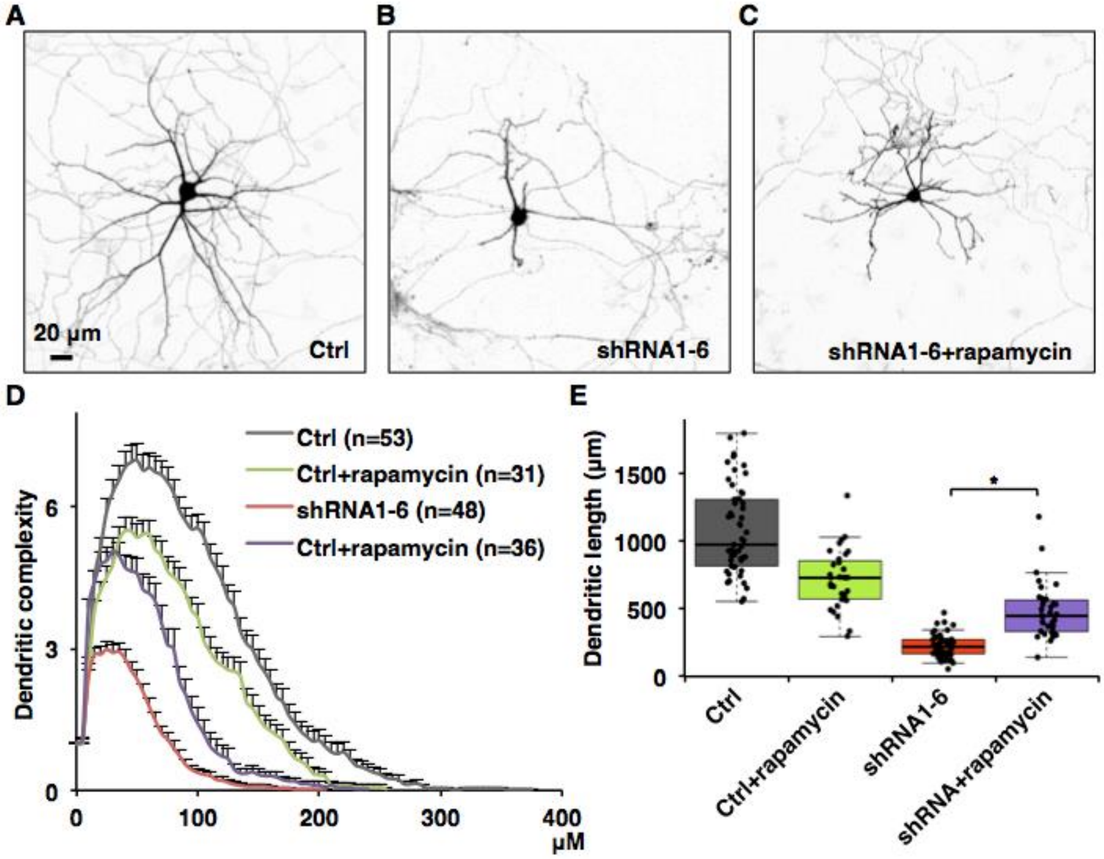
A
B
C
20 µm
Ctrl
shRNA1-6
shRNA1-6+rapamycin
D
E
Ctrl (n=53)
Ctrl+rapamycin (n=31)
shRNA1-6 (n=48)
Ctrl+rapamycin (n=36)
Dendritic complexity
0
3
6
0
100
200
300
400
µM
Dendritic length (µm)
0
500
1000
1500
*
Ctrl
Ctrl+rapamycin
shRNA1-6
shRNA+rapamycin

Figure S2.

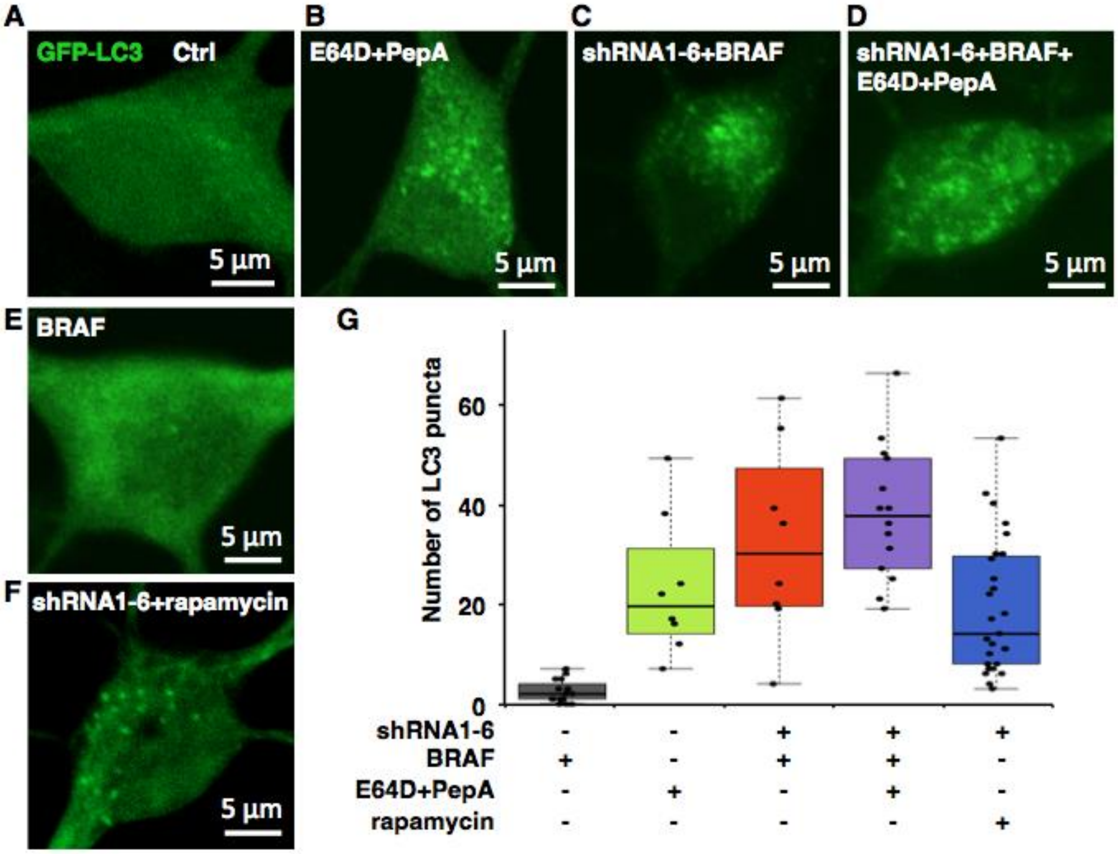
A
GFP-LC3 Ctrl
5 µm
B
E64D+PepA
5 µm
C
shRNA1-6+BRAF
5 µm
D
shRNA1-6+BRAF+
E64D+PepA
5 µm
E
BRAF
5 µm
F
shRNA1-6+rapamycin
5 µm
G
Number of LC3 puncta
0
20
40
60
shRNA1-6 - - + + +
BRAF + - + + -
E64D+PepA - + - + -
rapamycin - - - - +

Figure S3.

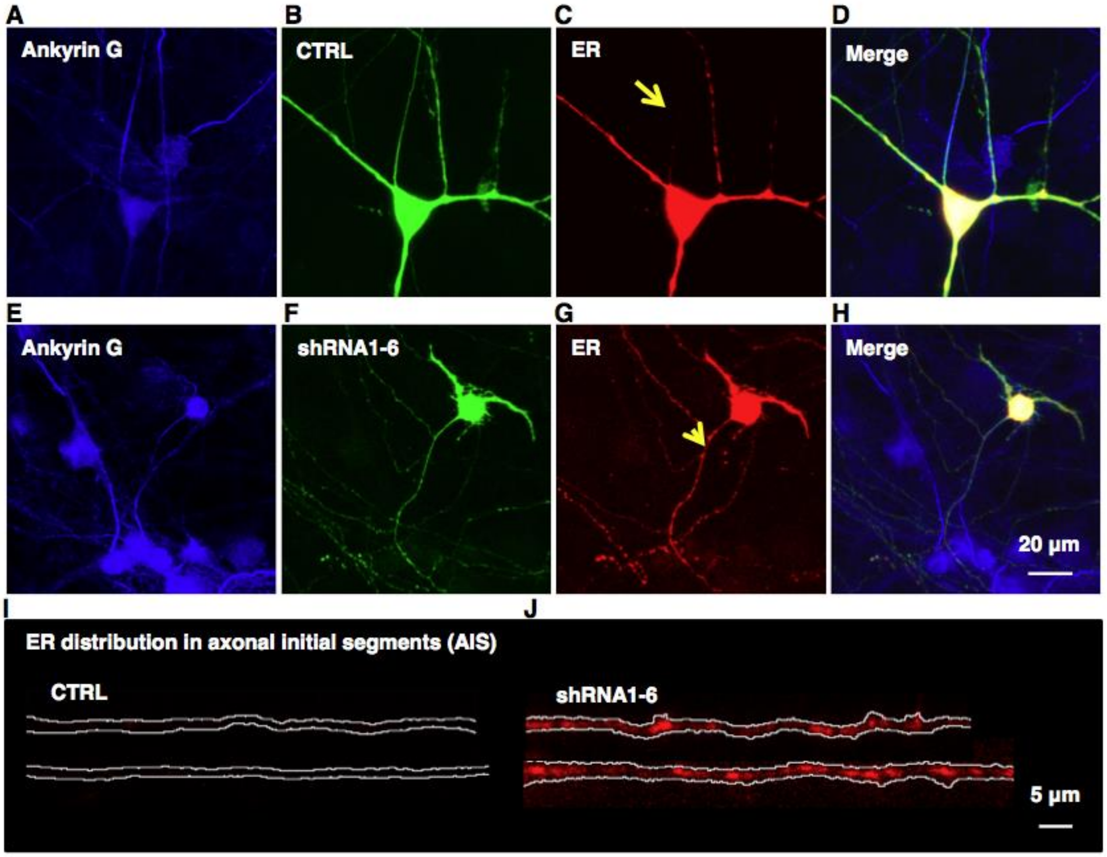
A
Ankyrin G
B
CTRL
C
ER
D
Merge
E
Ankyrin G
F
shRNA1-6
G
ER
H
Merge
20 µm
I
J
ER distribution in axonal initial segments (AIS)
CTRL
shRNA1-6
5 µm

Figure S4.

III. Supplemental Video Legends

Video 1. Representative Time-lapse Images of Dendritic Mitochondrial Motility in Control Neurons

Neurons were co-transfected at DIV6 with DsRed-mito (Red). Dendritic mitochondrial motility was observed in live neurons five days after transfection by time-lapse confocal microscopy using a laser-scanning confocal microscope (FV1000; Olympus). Frames were taken every five seconds for total 1000 seconds. Dendritic mitochondria show active movements in a saltatory manner: they frequently stopped and started to move in a single direction or reversed direction.

Video 2. Representative Time-lapse Images of Dendritic Mitochondrial Motility in DYNC1I1 Knockdown Neurons

Neurons were co-transfected at DIV6 with DsRed-mito (Red) and shRNA1-6. Dendritic mitochondrial motility was observed in live neurons five days after transfection by time-lapse confocal microscopy using a laser-scanning confocal microscope (FV1000; Olympus). Frames were taken every five seconds for total 1000 seconds. In contrast to the motility pattern found in the control neurons, the majority of dendritic mitochondria in DYNC1I1 Knockdown neurons are stationary, and only a few dendritic mitochondria are motile.

IV. Supplementary Experimental Procedures

Dynein intermediate chain gene formatting

We use standard gene formatting (Pfister et al., 2005): italicized initial uppercase followed by lowercase letters for rodents (*Dync1i1* and *Dync1i2*). For the formal names of proteins, the names in nonitalicized uppercase letters (DYNC1I1 and DYNC1I2) are used.

Plasmids

The constructs of Ras(G12V) and Myc-BRaf were gifted from Dr. Yan Chen (Institute for Nutritional Science, SIBS/CAS, Shanghai); the myr-Akt-delta4-129 plasmid was purchased from Addgene (Addgene ID: 10841); GFP-LC3 was gifted from Dr. Zun-ji Ke (Shanghai University of Traditional Chinese Medicine); ER-DsRed2 was gifted from Dr. Zheng Li (NIH, Maryland, MD); Ras(G12V) gene tagged with myc was subcloned into pcDNA3.1(-) vector (Invitrogen, Cat No: V795-20) with restriction enzyme sites EcoRI and BamHI; Ras mutants pcDNA3.1(-)-myc-Ras(G12V, T35S) and pcDNA3.1(-)-myc-Ras(G12V, Y40C) were mutated from pcDNA3.1(-)-myc-Ras(G12V). All constructs were confirmed by sequencing. *Dync1i1A* (GeneBank ID: KU343212), *Dync1i1B* (GeneBank ID: KU343213), *Dync1i1C* (GeneBank ID: KU343214), *Dync1i1D* (GeneBank ID: KU343215) were cloned from Sprague Dawley rat, the synthesized primers were:

Forward (F): 5' CGCGAATTCTGATGTCTGACAAGAGCGACCTAAAAGCTGAGC 3’
Reverse (R): 5’ TATGGATCCTGGGCAGCTAACTCAACCGCACCTTCCTC 3’

The amplified PCR fragments were cloned into the vector pEGFP-N1 with restriction sites EcoRI and BamHI (NEB).

Quantitative-PCR

Total RNA of Sprague Dawley P0 rat tissues were extracted and prepared with Trizol (Thermo Fisher, Cat No: 15596-026). First strand cDNAs were synthesised using oligo(dT)18 primers (Thermo Fisher, First Strand cDNA Synthesis Kit, Cat No: K1612) and RT-PCR reagent, which contains 4.6 μl cDNA (100 ng/μl), 0.2 μl each primer (10 μM) and 5 μl SYBR green mix (Thermo Fisher, Cat No: 4367659). The primers of *Dync1i1* and *Dync1i2* were listed as below:

Dync1i1	1A	Forward	5′-TGCAGCCGCTGCATTTTTTAACATG-3′
		Reverse	5′-GTTAATGGCCCCAGATCGTCTTGG-3′
	1B	Forward	5′-ATCGCCGGAGCCCCCTTTAGTCCCAAC-3′
		Reverse	5′-CTGGAGCACTGAGGGGTCTGTGTCCC-3′
	1C+1D	Forward	5′-GGGGCCATTAACAAGGAGAAGACTGAACAAG-3′
		Reverse	5′-CCTCATCTTCCTCATCCTCTTCTGACTG-3′
	1D	Forward	5′-TCTCTCCAGTACCCTGAGCTGATGG-3′
		Reverse	5′-ACACAGTACACAGGATGCTGACAGTGG-3′
Dync1i2	2A	Forward	5′-CCAATTGTTTTTTCTGAGCACTGGGTC-3′
		Reverse	5′-TAGGTCCTCGTCCCAAATCGGAATC-3′
	2B	Forward	5′-TCCCCAATTGTCCCTCCTCCCATG-3′
		Reverse	5′-TCGGAATCTGAGTGGAGCTGAAG-3′
	2C	Forward	5′-TCCCCAATTGTTCCTCCTCCCATG-3′
		Reverse	5′-GTTTAATAGGTCCTCGCCTAGATCCCAC-3′

All the reagents were pipetted into 384-well plate, gently spun down, and placed in 7500 Real-Time PCR System (ABI). Samples were Incubated at 95°C for 10 min, followed by 40 cycles of 95°C for 25 s, 60°C for 30 s, and 72°C for 30 s. Data were collected and analyzed by being normalized with GAPDH.

shRNA constructs:

The shRNA sequences of *Dync1li1* and *Dync1i2* were designed and listed as below：

Dync1i1	shRNA1-1	5′-GGACAATCGCAGTCATCGA-3′
	shRNA1-2	5′-GCAGTCAGACTCAGAACTT-3′
	shRNA1-3	5′-GGAGACACAGACTCCTCTT-3′
	shRNA1-4	5′-GCTGGAGCCAACCTTTCTT-3′
	shRNA1-5	5′-GCTGATGGTTGCTTCTTAT-3′
	shRNA1-6	5′-GCATGGAGCTGGTGTACAA-3′
	shRNA1-7	5′-GGTACCGTCTACACAGCTT-3′
Dync1i2	shRNA2-1	5′-GCAGGTGCTAAGCTGTCAT-3′
	shRNA2-2	5′-GCATGGAGTTGGTTCATAA-3′
	shRNA2-3	5′-GAAGAGAAGAGAAGCCGAA-3′

Forward oligos of shRNA include both sense and antisense orientational secquences, which are separated by a 9 bp spacer sequence. The 5' end contains a BglII site, while the 3' end has T5 sequence and a HindIII site. For example, the forward and reverse oligonucleotides of shRNA1-6 are designed as follows respectively:

5′ GATCCCC**GCATGGAGCTGGTGTACAA**TTCAAGAGA**TTGTACACCAGCTCCATGC**TTTTTA 3′
3′ GGG**CGTACCTCGACCACATGTT**AAGTTCTCT**AACATGTGGTCGAGGTACG**AAAAATTCGA 5′

Oligonucleotides were dissolved, mixed, and assembled by annealing. The modified pSUPER vector (*Kan*^{r}, EGFP expression) was linearized with BglII and HindIII restriction enzymes (NEB). The linearized vector and the annealed oligonucleotides were ligated (Takara, Ligation Kit Ver.2.1, Cat No: 6022) and transformed it into DH5α E. coli competent cells (Tiangen Biotech, Cat No: CB101-02).

The shRNA knockdown-resistant mutations of *Dync1i1* had six mutations in the nucleic acid sequences but no change in protein sequences. The mutation primers were as below:

F: 5' CCCCACAGGAGAGTATCGAACTCGTATATAACAAGTCCAAGCC 3'
R: 5' GGCAGCTAACTCAACCGCACCTTCCTC 3'

Western Blotting for shRNA efficiency

The efficiencies of shRNAs are analyzed with western blotting assay. Since DYNC1I1 only is expressed in brain, we transfected COS7, which has no endogenous DYNC1I1 expression, with *Dync1i1* and shRNA plasmids. COS7 cells were plated to 35mm dishes and co-transfected at 70-90% confluence with *Dync1i1* and shRNAs plasmids using Lipofectamine 2000 Transfection Reagent (Thermo Fisher, Cat No: 11668-019). Following transfection, cells were cultured for an additional 2 days before blotting. Cells were lysed with lysis buffer (1% Triton X-100 and 1% DOC in Tris-buffered saline buffer) supplemented with phosphatase and protease inhibitors. Protein extracts were resolved on 12% SDS-PAGE gel. The separated proteins were electrotransferred to Protran Nitrocellulose membranes (PerkinElmer). Membranes were blocked with 5% nonfat dry milk in Tris-buffered saline with 0.05% Tween 20 for 1 hour at room temperature. Membranes were then washed and incubated for overnight with GFP rabbit antibody (Abmart, 1:1000, Cat No: P30010) at 4 °C with gentle shaking. The membranes were washed and incubated for 1 h with anti-rabbit second antibody (GE, 1:1000, Cat No: NA934) at room temperature. The immunoblots were visualized with ECL Western Blotting Substrate (Pierce, Cat No: 32109) with exposure of the blots to X-OMAT BT film.

Hippocampal Neuron Culture and Transfection

Hippocampi were dissected from postnatal day 1 Sprague Dawley rat pups and cultured as described previously (Kang et al., 2008). Briefly, after digestion with trypsin and triturating with fire-polish pipettes, the hippocampal cells recovered by centrifugation were plated onto 12 mm coverslips, and after 2 hours, 2 ml plating medium was added to each 35 mm dish [For 100 ml of plating medium: 89 ml Minimal Essential Medium (Invitrogen), 0.5 g glucose, 0.5 mM glutamine, 2 g $NaHCO_2$, 10 mg bovine transferrin (Calbiochem), 2.5 mg insulin, 10% fetal calf serum]. From

the second day in culture, half of the medium was replaced with feeding medium twice a week [100 ml of feeding medium: 97 ml minimal essential medium, 0.5 g glucose, 0.5 mM glutamine, 2 g $NaHCO_2$, 10 mg bovine transferring, 3 mM cytosine-p-arabinofuranoside (Sigma-Adrich) and 2% B27 medium supplement (Invitrogen)]. For immunocytochemistry or live imaging, a low density (6000-8000 cells per cm^2) of neurons was cultured on Matrigel (BD Biosciences) coated coverslips from hippocampi of postnatal day 1 rat pups. Neuronal transfection was performed at 6 days *in vitro* using the calcium phosphate method. Following transfection, cells were cultured for an additional 5 days before imaging or immunocytochemistry.

Imaging and Immunocytochemistry

All imaging was performed using an Olympus FV1000 confocal microscope with a 40×/0.95 objective (Olympus) for dendritic length imaging and a 60×/1.2w objective (Olympus) for high-resolution imaging at room temperature or mitochondrial motility study at 37 °C. Cell cultures were fixed with 4% paraformaldehyde and 4% sucrose in 1x PBS, and permeabilized with 0.5% Triton X-100 for 30 min at 22-24°C. Primary antibodies against dendritic marker MAP2 (BD Pharmingen, mouse, Cat No: 556320), axonal marker neurofilament (Millipore, chicken, Cat No: AB5539), AIS marker Ankyrin G (Santa Cruz, rabbit, Cat No: sc-28561) and myc tag (Abmart, mouse, Cat No: m20002s) were applied, followed by washes in 1x PBS and visualization with Alexa 488-, 546-, or 633-conjugated secondary antibodies (Invitrogen). To block nonspecific signals, 5% or 2% goat serum was added during the incubation with primary or secondary antibodies, respectively. The immunostaining with myc antibody was used to confirm the expression of BRAF and RAS mutants after transfection.

Kymographs

Kymographs were made as described previously (Kang et al., 2008) with extra plug-ins for ImageJ (NIH). Briefly, we used plug-ins: “Straighten” to straighten curved neurites, “Grouped_ZProjector” to z-axially project re-sliced time-lapse images, “Time_Stamper” to make time-stamped images/frames. The height of the kymographs represents recording time (1000 seconds) and the width of the kymographs represents length (μm) of the axon imaged.

Data Quantification for Mitochondrial Motility Study

Time-lapse imaging was performed in the modified Tyrode's solution (in mM: 10 Hepes, 10 glucose, 3 KCl, 145 NaCl, 1.2 CaCl2, 1.2 MgCl2, and 1 μM glycine, pH 7.4). For imaging live neurons with reasonable signal-to-noise ratio, the lowest intensity of lasers and the shortest scanning time were used to minimize bleaching and damage to neurons; pinhole were opened to allow signals of interest to be collected. Time-lapse images were collected around 512 x 512 pixel resolution (12 bit). To trace motile mitochondria in dendrites, five-seconds interval for a total of 200 images was used to minimize laser-induced cellular damage unless otherwise noted.

To quantify the motility of dendritic mitochondria, motile and stationary dendritic mitochondria are separated with fast Fourier transform (FFT) algorithm. Considering motile mitochondria is prone to be ambiguous and overestimated due to the vague definition of motile mitochondria and the effect of photobleaching, we have quantified and compared the absolutely stationary mitochondria. Kymographs were used to trace the movement of mitochondria and visualize stationary ones. After background being substracted, ratiometric intensity values of stationary kymographs to the sum values of motile and stationary kymographs were calculated per experiment for the percentage of stationary dendritic mitochondria.

The mobile and stationary components of time series images are separated with an immobile filter, which is computed with FFT algorithm using MATLAB (Mathworks). Each pixel of time-series images is applied the FFT processing to obtain the series in frequency domain of each pixel. The mobile components in time domain are the real part of inverse FFT of the series in frequency domain after the first item of the frequency-domain series is set to be zero, while the stationary component in time domain is the real part of inverse FFT of the series in frequency domain after the rest items except the first item of the frequency-domain series are set to be zero.

MATLAB source code of immobile filter is included as below:

```
%----------------The beginning of the source ------------------
```

```
function [mobile,immobile] = immobfilter_dtft( movie )

siz_mov = size(movie);
moviefft = fft(movie,[],3);

mobilefft = moviefft;
mobilefft(:,:,1) = 0;
mobile = real(ifft(mobilefft,[],3));

immobilefft = moviefft;
immobilefft(:,:,2:siz_mov(3)) = 0;
immobile  = real(ifft(immobilefft,[],3));
%-----------------The end of the source-------------------
```

V. Supplemental References:

Kang, J.-S., Tian, J.-H., Pan, P.-Y., Zald, P., Li, C., Deng, C., and Sheng, Z.-H. (2008). Docking of Axonal Mitochondria by Syntaphilin Controls Their Mobility and Affects Short-Term Facilitation. Cell *132*, 137–148.

Pfister, K.K., Fisher, E.M.C., Gibbons, I.R., Hays, T.S., Holzbaur, E.L.F., McIntosh, J.R., Porter, M.E., Schroer, T.A., Vaughan, K.T., Witman, G.B., et al. (2005). Cytoplasmic dynein nomenclature. J Cell Biol *171*, 411–413.
